# PD-L1 Regulates Platelet Activation and Thrombosis *via* Caspase-3/GSDME Pathway

**DOI:** 10.3389/fphar.2022.921414

**Published:** 2022-06-15

**Authors:** Yulong Li, Guang Xin, Shiyi Li, Yuman Dong, Yuda Zhu, Xiuxian Yu, Chengyu Wan, Fan Li, Zeliang Wei, Yilan Wang, Kun Zhang, Qingqiu Chen, Hai Niu, Wen Huang

**Affiliations:** Laboratory of Ethnopharmacology, Tissue-orientated Property of Chinese Medicine Key Laboratory of Sichuan Province, West China School of Medicine, West China Hospital, Sichuan University, Chengdu, China

**Keywords:** platelet, PD-L1, stroke, aggregation, thrombosis

## Abstract

Platelets play a central role in hemostasis and thrombosis, regulating the occurrence and development of thrombotic diseases, including ischemic stroke. Programmed death ligand 1 (PD-L1) has recently been detected in platelet, while the function of PD-L1 in platelets remain elusive. Our data reveal a novel mechanism for the role of PD-L1 on platelet activation and arterial thrombosis. PD-L1 knockout does not affect platelet morphology, count, and mean volume under homeostasis and without risk of bleeding, which inhibits platelet activation by suppressing outside-in-activation of integrin by downregulating the Caspase-3/GSDME pathway. Platelet adoptive transfer experiments demonstrate that PD-L1 knockout inhibits thrombosis. And the absence of PD-L1 improves ischemic stroke severity and increases mice survival. Immunohistochemical staining of the internal structure of the thrombus proves that PD-L1 enhances the seriousness of the thrombus by inhibiting platelet activation. This work reveals a regulatory role of PD-L1 on platelet activation and thrombosis while providing novel platelet intervention strategies to prevent thrombosis.

## Introduction

Thrombotic diseases have been increasing in recent years. The morbidity and mortality of ischemic stroke caused by thromboembolic events have gradually increased, and it has gradually become one of the leading causes of death (Wang W. et al., 2017; Collaborators, 2021). Ischemic stroke has an apparent prethrombotic state, mainly characterized by abnormal platelet activation (Langer and Chavakis, 2013; Staessens et al., 2020). Platelets are small non-nucleated cell elements produced in the bone marrow from megakaryocytes (van der Meijden and Heemskerk, 2019), maintaining normal blood flow and stopping bleeding. When the vascular wall is damaged or stimulated by chronic inflammation, platelets are rapidly activated and release a large number of related molecules, act on platelet receptors to initiate intra-platelet signaling pathways, which shift platelet integrin αIIbβ3 from a low-to a high-affinity state and enable platelet aggregation and thrombus formation through binding of soluble fibrinogen and other αIIbβ3 ligands (Hynes, 1992). Ligand binding to αIIbβ3 also triggers αIIbβ3 outside-in signaling (Calaminus et al., 2007; Li et al., 2010), leading to tyrosine phosphorylation of signaling proteins (Wonerow et al., 2003), including C-terminal Src kinase (c-Src) and phospholipase Cγ2 (PLCγ2), initiating downstream platelet responses, such as granule secretion, platelet spreading and clot retraction (Watson et al., 2005; Klarström Engström et al., 2014). Thus, activation of platelet integrin receptors is essential for hemostasis and thrombosis.

Programmed death ligand-1 (PD-L1), also known as CD279 or B7 - H1, is a type 1 transmembrane glycoprotein that belongs to the family B7 (Bodhankar et al., 2013; Ohaegbulam et al., 2015; Feng et al., 2019). PD-L1 has been shown to play a well-characterized role in inhibiting antitumor immunity, but little is known about the non-immunoregulatory function of PD-L1. Recently, the research found that human and mouse platelets detect PD-L1 (Zaslavsky et al., 2020; Hinterleitner et al., 2021). And platelet CD62P expression levels showed a strong positive correlation with PD-L1 expression (Hinterleitner et al., 2021), suggesting that PD-L1 correlates with platelet activation status. Bodhankar and colleagues found that PD-L1 may be related to ischemic stroke (Bodhankar et al., 2013; Bodhankar et al., 2015), but the role of PD-L1 in stroke has not yet been explored.

Here, we explored the effect of PD-L1 on platelet function and subsequent thrombosis and ischemic stroke. We show that inhibition of PD-L1 can suppress platelet activation and improve the formation of arterial thrombosis and stroke by downregulating the Caspase-3/GSDME pathway, suggesting the possibility of targeting PD-L1 as a potential approach for interfering with thrombosis without risk of bleeding.

## Materials and Methods

### Animals

8-week-old SPF male C57BL/6 mice (Chengdu Dashuo Experimental Animal Co., Ltd. license number: SCXKchuan 2015-030), 25 ± 5 g, animal experiments, conventional knockout model of PD-L1^−/−^ mice are from the Department of Gastrointestinal Surgery, West China Hospital, Sichuan University, Professor Yuan Li, and methods were approved by the Ethics Committee of West China Hospital of Sichuan University. In our study, we used the conventional PD-L1 knockout mice, the mice strain is C57BL/6 mouse, knockout gene name (NCBI number): 60533, knockout gene name (MGI number): CD274 (MGI: 1926446) knockout gene sequence; r = 19: 29367455-29388095; t = ENSMUST00000016640. Mice Gene Identification Sequence as follows: PD-L1 KO CD274-KO-tF1 (CCA​CAG​GAG​ACA​GTT​TGG​TGA​GAG​G), Gps00000935-CD274-KO-tR1 (GGC​TTC​CAC​CAC​CAA​AGT​GTT​T). We have defined untreated Wild-type mice platelet as controls in this study.

### Materials

Antibodies against PD-L1 and Caspase-3 (Abcam, United States). Cleaved Caspase-3, and c-Src (anti-Tyr-416) (Cell Signaling Technology, United States); PLCγ2 (anti-Tyr-1217) and pan-PLCγ2 (Bioworld Technology, United States), Thrombin and fibrillar type I collagen was purchased from Sigma. Protein concentration was quantified using a BCA protein assay kit (Beyotime Biotechnology China); pan-c Src, Goat anti-rabbit IgG-HRP and goat anti-mouse IgG-HRP (Proteintech, China). Recombinant human IL-1β (R&D Systems,United States). JON/A-PE and R300 (emfret, Germany). CD62P (P-Selectin) Monoclonal Antibody (Psel.KO2.3), PE, eBioscience™ (Thermo Fisher, United States).

### Platelet Preparation

Blood was collected from pentobarbital-narcosed mice from the heart into 3.8% citrate anticoagulant. Washed platelet was prepared as previously described (Munzer et al., 2017). Platelet-rich plasma (PRP) was obtained by centrifugation at 260 *g* for 5 min and used for aggregation tests (Guo et al., 2017). Afterward, PRP was centrifuged at 600 g for 10 min to pellet the platelet. To ease platelets prostaglandin I_2_ (0.5 µM, Merck Calbiochem) were added to the PRP, after washing steps, the pellet of washed platelets was resuspended in PBS. Platelet counts were normalized and used for spreading (2 × 10^7^/ml) or biochemical analysis (4–5 × 10^8^/ml).

### Platelet Aggregation Tests

Platelet-rich plasma (PRP) from Wild-Type (WT) and PD-L1 KO mice were stirred (1,200 rpm) at 37°C in a platelet aggregation analyzer (AG400) before the addition of agonists (thrombin, collagen, adenosine diphosphate or U46619). Aggregation was measured as percent change in light transmission for 5 min (Guo et al., 2017; Zhou et al., 2020).

### Flow Cytometry

Platelet from mice was incubated with JON/A, and CD62P for 20 min in the dark at room temperature, then stimulated with thrombin (Thr 0.05 U/mL) (Södergren and Ramström, 2018). Surface expression of platelet-specific receptor GPVI and αIIBβ3 was measured using anti-human Glycoprotein VI purified antibody (followed by addition of FITCconjugated goat anti-mouse IgG) and FITC-conjugated mouse anti-human CD41a antibody respectively. Samples were run using a FACScan (BD Biosciences). Data were analyzed using FlowJo v10 software.

### Platelet Spreading

Platelet (2 × 10^7^/ml) were placed on fibrinogen-coated glass coverslips (100 μg/ml fibrinogen, 4 °C overnight) at 37 °C for 90 min (Hosseini et al., 2019; Zhou et al., 2020). After washing with PBS, the platelet was fixed, permeabilized, stained with Alexa Fluor-546-labelled phalloidin, and viewed by fluorescence microscopy (Nikon-80i) using an ×100 oil objective. Surface coverage and the number of platelet adhesion on fibrinogen were quantified using ImageJ software.

### Platelet Spreading and Adhesion Assay

Washed platelets (2 × 10^7^/ml) incubated with homologous or heterologous poor platelet plasma for 1.5 h. Washed platelets again and labeled them with PKH26 for 20 min. Then platelets were placed on fibrinogen-coated glass coverslips (100 μg/ml fibrinogen, 4 °C overnight) at 37 °C for 90 min (Xin et al., 2016). Platelet viewed by fluorescence microscopy (Nikon-80i) using an ×100 oil objective. Surface coverage and the number of platelet adhesion on fibrinogen were quantified using ImageJ software.

### Transmission Electron Microscopy Imaging of Platelet

Washed platelet was fixed in 3% glutaraldehyde in 0.1 M sodium cacodylate buffer (pH 7.4), dehydrated with a graded series of ethanol. The sample was dropped on a 6 mm glass slide, sprayed with gold, and dried (Graham and Orenstein, 2007). The specimens were visualized in a JEM transmission electron microscope (EVO 10).

### Clot Retraction Tests

Platelet (3 × 10^8^/ml) were supplemented with 2 mM Ca^2+^ and 0.5 mg/ml fibrinogen and clot retraction were initiated by thrombin (1 U/ml) stimulation at 37 °C (Brzoska et al., 2013). Images were captured every 10 min.

### Western Blot Analysis

Total proteins extracted from platelet using RIPA lysis buffer. The concentrations of proteins were detected using a BCA kit (Beyotime Biotechnology). Platelet proteins were separated on SDS-PAGE gels and transferred to PVDF membranes (Fang et al., 2018). To evaluate proteins levels, the blots were blocked with 5% non-fat milk in TBS with Tween-20 at room temperature for 1 h and incubated with primary antibodies PD-L1 (1:1,000, Abcam), Caspase-3 (1:1,000, Abcam), Cleaved Caspase-3 (1:1,000, Cell Signaling Technology), GAPDH was used as the internal control. The blots were incubated with different dilutions of primary and secondary Abs. Ab binding was detected using enhanced chemifluorescence (ECF; GE Healthcare). The blots visualized use the Odyssey Fc System (LI-COR Biosciences). Densitometry of the protein bands was performed using Image lab software. The experiments were repeated at least three times.

### FeCl_3_-Induced Thrombosis Model

For the FeCl_3_-induced thrombosis model, exogenous carboxyfluorescein succinimidyl ester (CFSE)–labeled platelet was infused into the tail vein. Mice were anaesthetized using 1% pentobarbital, then the common carotid artery was carefully exposed and kept moist by super-fusion with warm (∼37°C) saline, a filter paper (5 × 3 mm) saturated with 5% ferric chloride (FeCl_3_) solution was applied topically for 1 min, the thrombus formation in the injured carotid vessel was monitored using a Nikon A1RMP + Two-Photon Microscope (Nayak et al., 2021), Time to occlusion exceeding 30 min was noted as 30 min. The time to form occlusive thrombus was considered as the time required for blood to stop flowing completely for >1 min. In adoptive transfer experiments, Wild-Type (WT) and PD-L1 KO platelets were isolated and labeled with CFSE. The recipient WT mice or PD-L1^−/−^ mice were pretreated with an anti-CD42b antibody (R300, 0.5 μg/g body weight) to deplete endogenous platelets, 30 minutes after antibody treatment, the CFSE-labeled WT or PD-L1 KO platelets, 10^8^ in 100 µL of saline, were infused into the thrombocytopenic PD-L1 KO or WT mice (Kim et al., 2017). Afterwards, the same experimental method was used to record the process of 5% FeCl_3_ induced carotid artery thrombosis and analyze the time of vessel occlusion, as described above.

### Tail Bleeding Time Tests

We used mice (10 ± 2 weeks old) of the same weight (25 ± 3 g) anesthetized with pentobarbital 0.4 mg/g intraperitoneally (i.p.). In the transaction model, tails were cut 2 mm from the tip with a razor blade, and the tails were immediately immersed in 37 °C salines, and the time to cessation of bleeding was recorded (Cardenas et al., 2018). All animals were hemostasis after bleeding at 12 min.

### tMCAO Model

Transient middle cerebral artery occlusion (tMCAO) was performed as described previously (Liu et al., 2016). Anesthesia was induced by inhalation of 5% isoflurane and maintained by intraperitoneal injection of 1% pentobarbital. After a midline incision in the neck, the proximal common carotid artery and the external carotid artery were ligated, and a standardized silicon rubber-coated 6.0 nylon monofilament (Doccol Corp, Redlands, CA) was inserted and advanced via the right internal carotid artery to occlude the origin of the right MCA. The intraluminal suture was left *in situ* for 60 min. Next, monofilament was withdrawn to allow reperfusion.

### Neurological Tests

24 h after induction of tMCAO, the Bederson test was used to assess global neurological (Bieber et al., 2019).

### Cerebral Lesion Quantification

To measure cerebral infarct volumes, mice were euthanized 24 h after induction of MCAO. Using a mouse brain slice matrix, brains were quickly isolated and cut into 2-mm-thick coronal sections. The slices were stained with 2% 2,3,5-triphenyl-tetrazolium chloride (TTC, Sigma Aldrich, Saint Louis, MO) to distinguish healthy from infarcted tissue. Stained slices were photographed, and infarct areas (white) were quantified using ImageJ software (Denorme et al., 2020).

### Thrombus Histology

Thrombi were gently removed from the carotid artery or brain tissue, washed in saline, and immediately incubated in 4% paraformaldehyde for 24 h at room temperature. Next, samples were embedded in paraffin and cut into 5 mm sections (Kwon et al., 2013). Thrombus sections were stained with Anti-CD42b (ab183345, Abcam). And the platelet was stained brown. Brain tissue sections were stained with H&E (HT110216, Sigma-Aldrich). Images from immunofluorescent stainings were acquired using an Axio Observer Z1 inverted fluorescent microscope. Images were processed by Zen 2012 (blue edition, version 2.3, Zeiss) software.

### Statistical Analysis

All data are presented as mean ± standard deviation (SD). For statistical analysis, Prism 8 software (GraphPad) was used. The unpaired 2-tailed Student’s *t-test* was used to compare experiments with two groups. One-way ANOVA with Bonferroni’s test was used for data comparison when comparing three or more experimental groups, and two-way ANOVA with Bonferroni’s test was performed for comparison among multiple groups. *p* values less than 0.05 were considered significant.

## Results

### PD-L1 Knockout Does Not Affect Platelet Parameters, Morphology and Ultrastructure Without Stimulation

**FIGURE 1:**
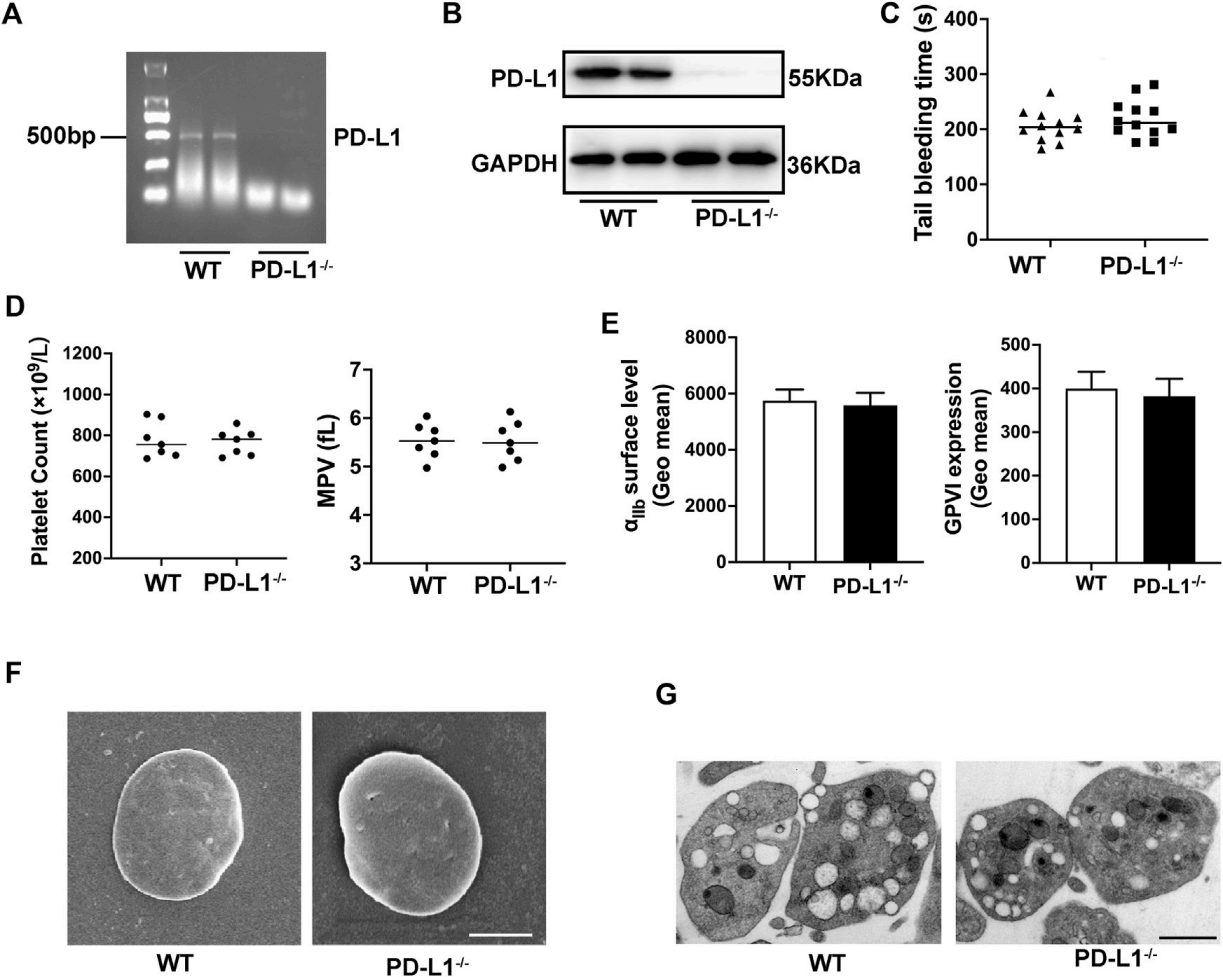
PD-L1 knockout does not affect platelet count, volume, morphology, and ultrastructure under homeostasis. **(A)** PD-L1 DNA expression in platelet from PD-L1^−/−^ mice; *n* = 5. **(B)** Representatives immunoblot of the PD-L1 protein in platelet from WT mice and PD-L1^−/−^ mice; *n* = 5. **(C)** Tail bleeding time measured after amputating 2 mm of the tail tip. Each dot represents 1 individual mice. *n* = 12. **(D)** Platelets count and mean platelets volume (MPV) were determined from PD-L1^−/−^ and WT mice by an automatic blood analyzer; *n* = 7. **(E)** The expression of platelet receptors αIIbβ3 and GPVI was detected by flow cytometry, *n* = 7. **(F)** Representative scanning electron microscope images of platelets morphology from WT mice and PD-L1^−/−^ mice; Scale bar = 1 μm; *n* = 5. **(G)** Representative transmission electron microscopy images of platelets ultrastructure from WT mice and PD-L1^−/−^ mice. Scale bar = 1 μm; *n* = 5. Data are mean ± SD.

To explore the role of PD-L1 in platelet, we generate PD-L1 knockout mice. Quantitate Real-time polymerase chain reaction analysis (RT-PCR) result showed that PD-L1 expression is wholly ablated in PD-L1 knockout mice platelets (Figure 1A). Indeed, immunoblotting also proved the absence of PD-L1 in PD-L1 knockout platelets (Figure 1B). However, impaired platelet function would reduce the hemostatic capacity of platelets and increase the risk of bleeding. The tail bleeding time test showed that PD-L1^−/−^ mice had a normal bleeding time compared with wild-type (WT) mice (Figure 1C), which indicated that PD-L1 was a safe target in circulating blood without the risk of bleeding. Of note, the platelet count is also one of the determinants of bleeding risk (Vinholt, 2019). Our results showed a similar value of platelet count and mean platelet volume in WT and PD-L1^−/−^ mice (Figure 1D). Platelet receptors GPVI and integrin αIIbβ3 are critical for platelet function (Swieringa et al., 2014). We evaluated these receptors by flow cytometry, and our results revealed equivalent GPVI and integrin αIIbβ3 receptor protein levels in WT and PD-L1^−/−^ mice (Figure 1G). Electron microscopy analysis showed that PD-L1 KO did not affect platelet disc morphology and ultrastructure (Figures 1E,F). Together, these data suggested that PD-L1 did not affect platelet count, expression of platelet receptors, or ultrastructure in unstimulated circulating blood.

### PD-L1 Knockout Inhibits Platelets Aggregation in Response to Different Agonists

**FIGURE 2:**
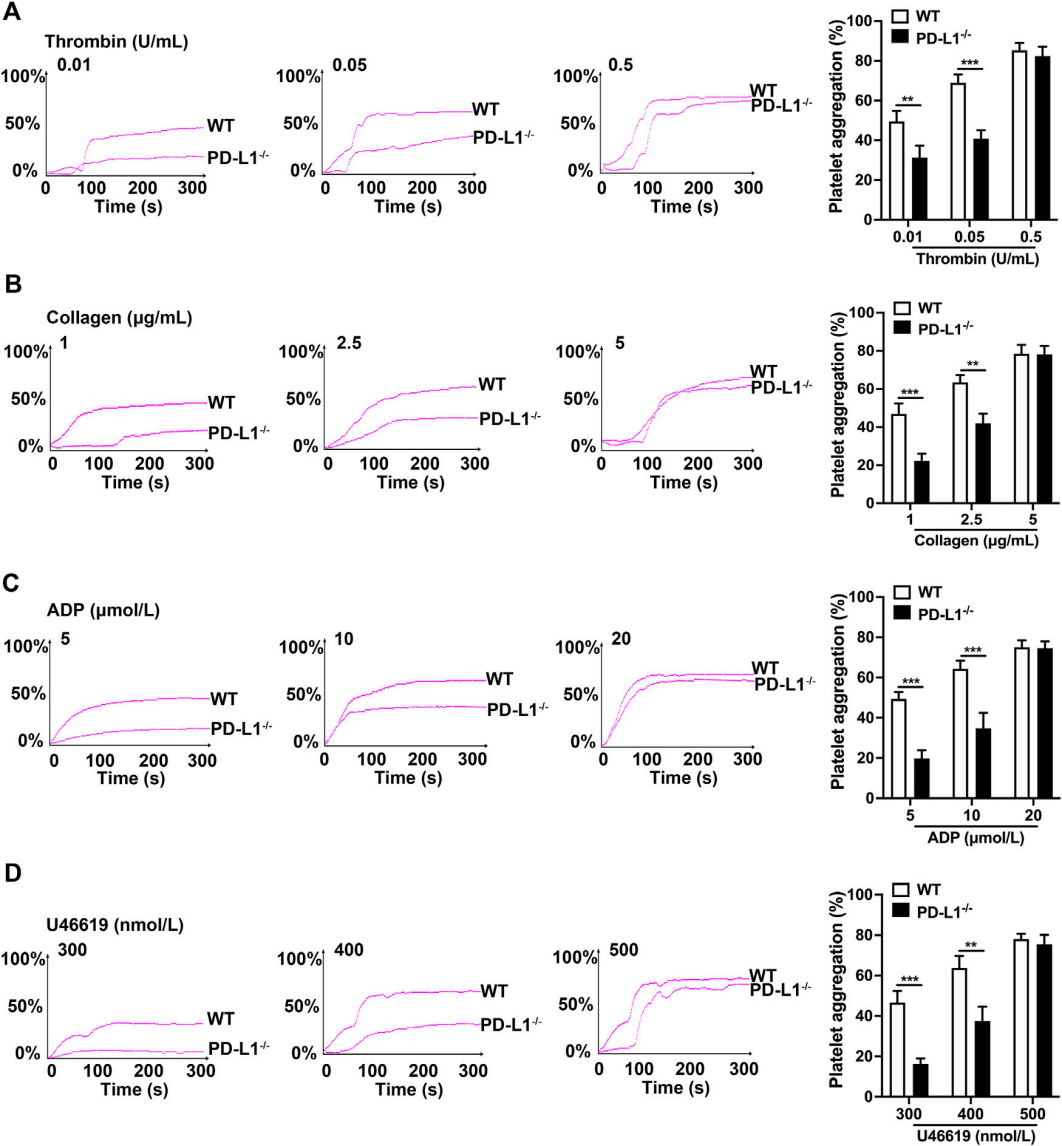
PD-L1 knockout reduces platelets aggregation in responded to the stimulations of thrombin, ADP, collagen, and U46619. Platelet-rich plasma from WT or PD-L1^−/−^ mice were stimulated with different concentrations of thrombin (0.01, 0.05 or 0.5 U/ml) **(A)**, collagen (1, 2.5 or 5 μg/ml) **(B)**, ADP (5, 10 or 20 μmol/ml) **(C)**, and U46619 (300, 400 or 500 nmol/ml) **(D)** at 37 °C to detect aggregation using the AG400 semi-automatic platelet aggregation analyzer for 5 min. Aggregation was expressed as the maximal percentage of light transmitted. The graphs are representative experiments. In the histograms of maximal platelets aggregation under the indicated conditions. Data are mean ± SD; *n* = 5, **p < 0.01, ***p < 0.001.

In blood circulation, platelets can quickly adhere and aggregate at sites of vascular injury, which is the initial stage of thrombosis (Hou et al., 2015; Xu et al., 2016; Rana et al., 2019). Therefore, we investigated the effect of PD-L1 on platelet aggregation. Our results show that the aggregation of PD-L1 KO platelets was significantly diminished, compared with that of WT platelet, in response to a low and higher dose of thrombin (0.01 and 0.05 U/ml) (Figure 2A), collagen (1 and 2.5 μg/ml) (Figure 2B), ADP (5 and 10 μmol/ml) (Figure 2C), and U46619 (300 and 400 nmol/ml) (Figure 2D). However, no differences in platelet aggregation were found in response to a relatively high dose of thrombin (0.5 U/mL), collagen (5 μg/ml), ADP (20 μmol/ml) as well as U46619 (500 nmol/L). These results suggested a contributory role of PD-L1 in platelet aggregation.

### PD-L1 Modulates Platelets Integrin αIIbβ3 Outside-in Activation

**FIGURE 3:**
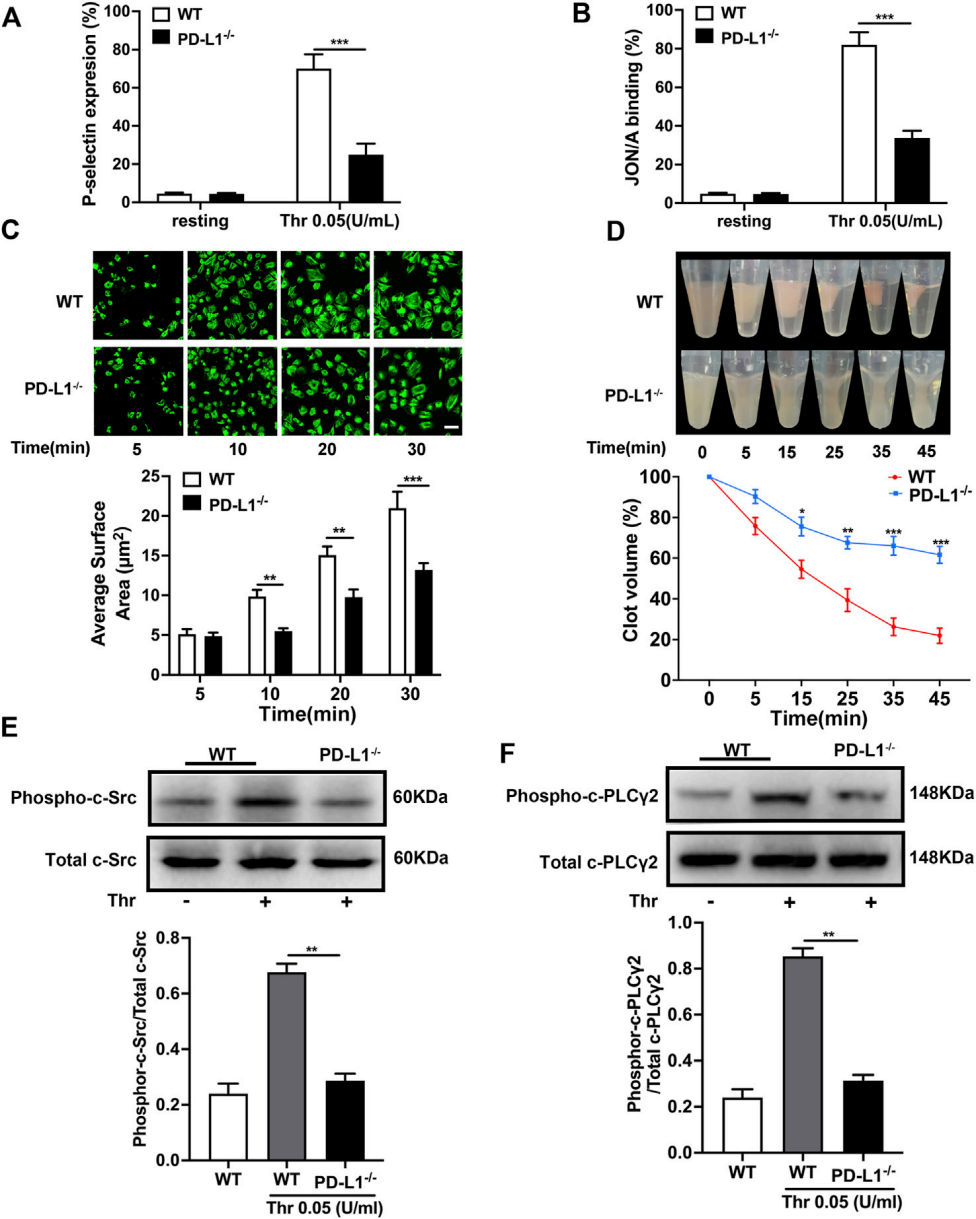
PD-L1 knockout inhibites platelet integrin αIIbβ3 outside-in signaling. **(A)** Flow cytometry analysis of FITC-CD62P binding to resting platelets or platelets stimulated with thrombin (0.05 U/mL) for 5 min. **(B)** Flow cytometry analysis of PE-JON/A binding to resting platelets or platelets stimulated with thrombin (0.05 U/mL) for 5 min. **(C)** Washed platelets were allowed to adhere and spread on fibrinogen-coated wells by thrombin (0.05 U/mL) stimulation at 37 °C for 5, 10, 20, 30 min. The spreading area of single platelet was measured using ImageJ2x software, with pixel number as the unit of size. **(D)** Clot retraction was measured for 45 min in PRP, after adding 0.25 U/mL of thrombin and incubation at 37 °C. Washed platelets in modified thyroid’s buffer were mixed with 100 μg/ml purified human fibrinogen. **(E)** Representatives immunoblot of the phosphorylation of c-Src in platelet from WT mice and PD-L1^−/−^ mice. **(F)** Representatives immunoblot of the phosphorylation of PLCγ2 in platelet from WT mice and PD-L1^−/−^ mice. Data are mean ± SD; *n* = 5. *p < 0.05, **p < 0.01, ***p < 0.001.

Platelet granule secretion induced by agonist stimulation plays a critical role in amplifying platelet signaling and subsequent activation and aggregation (Zhou et al., 2020). We measured platelet degranulation by flow cytometry, represented by P-selectin expression and αIIbβ3 activation. As expected, the expression of P-selectin and activation of integrin αIIbβ3 in PD-L1 KO platelets were significantly reduced compared with WT mice platelets (Figures 3A,B). And platelet spreading and clot retraction, two processes regulated by αIIbβ3 outside-in signaling, also weakened in PD-L1^−/−^ mice, respectively (Figures 3C,D). These results suggested that PD-L1 might be involved in integrin signaling regulation. Indeed, immunoblotting showed that PD-L1 knockout reduced the expression of phosphorylation of c-Src (Figure 3E) and phosphorylation of PLCγ2 (Figure 3F) in platelet, which mediated the integrins activation of outside-in signaling. Our results showed that inhibition of PD-L1 attenuates the outside-in activation of platelet integrin αIIbβ3 resulting in damaged platelet clots forming. Our results showed that knockout of PD-L1 inhibited integrin-αIIbβ3-mediated outside-in signaling, an amplifier of platelet activation, and inhibited platelet clot formation and retraction.

### PD-L1 Regulates Platelet Activation by Downregulating the Caspase-3/GSDME Pathway

**FIGURE 4:**
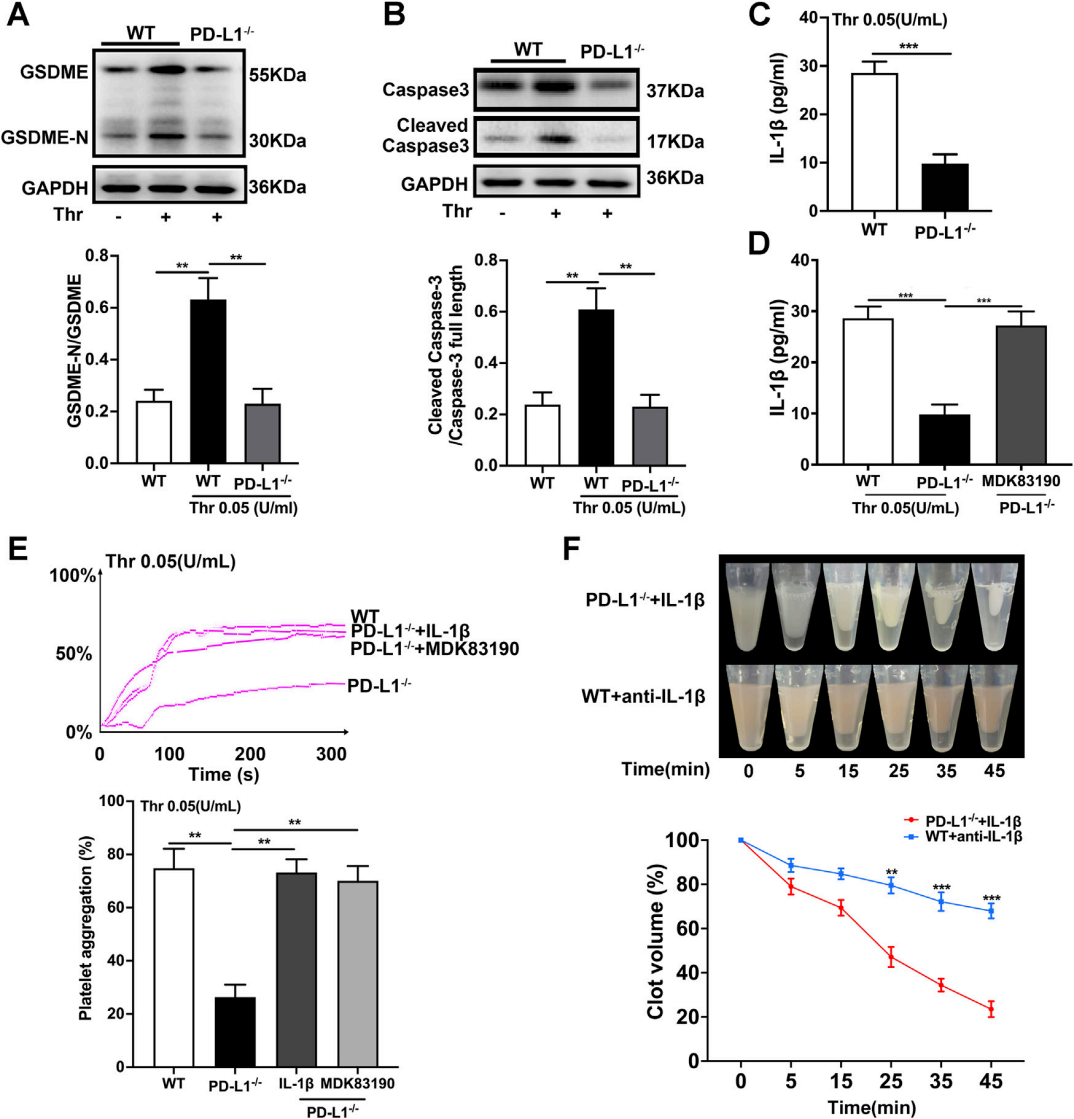
PD-L1 knockout regulates platelet activation by downregulating Caspase-3/GSDME pathway. **(A)** Representatives immunoblot of the GSDME in platelets of WT mice and PD-L1^−/−^ mice. GAPDH was used as loading controls. **(B)** Representatives immunoblot of the Caspase-3 in platelet from WT mice and PD-L1^−/−^ mice. GAPDH was used as loading controls. **(C)** The levels of platelets IL-1β in WT and PD-L1 KO platelet detected by enzyme-linked immunosorbent assay. **(D)** The levels of platelets IL-1β in MDK83190 treated PD-L1 KO platelet detected by enzyme-linked immunosorbent assay. **(E)** PRP from PD-L1^−/−^ mice were pre-treated IL-1β or MDK83190 for 1.5 h with stimulation by thrombin (0.05 U/ml) at 37 °C. Aggregation was expressed as the maximal percentage of light transmitted. In the histograms of maximal platelets aggregation under the indicated conditions. **(F)** Clot retraction was studied using washed platelets treated with 0.25 U/mL thrombin in the presence of 10 ng/ml recombinant mouse IL-1β or 0.5 μg/ml anti-IL-1β antibody at 37 °C. Representative images at 0, 5, 15, 25, 35 and 45 min. Dates were quantified as the clot volume (%) and are presented as mean values (two-way ANOVA). Data are mean ± SD; *n* = 5. **p < 0.01, ***p < 0.001.

PD-L1 participates in the regulation of GSDME activation in melanoma cells (Santarpia et al., 2020). And the function of GSDME in platelet remain elusive. Therefore, we assessed the effect of PD-L1on platelet GSDME. In the PD-L1 KO platelet, the expression of GSDME was significantly inhibited (Figure 4A). And the expression of Caspase-3 was also decreased (Figure 4B), which is responsible for the regulation of GSDME (Jiang et al., 2020). Recent studies identified GSDME as a conduit for IL-1β release (Zhou and Abbott, 2021) that allows IL-1β release and membrane leakage. To ensure the regulation of PD-L1 on platelet GSDME, we conducted ELISA on platelet IL-1β release. ELISA data confirmed that the release of IL-1β from PD-L1 KO platelets is more minor than in WT mice (Figure 4C). To further verify this result, we analyzed the IL-1β release in the PD-L1 KO platelet treated by the agonist MDK83190 of Caspase-3, which showed a significant increase (Figure 4D). Our data suggest that PD-L1 knockout downregulated Caspase-3/GSDME signaling repressing the release of IL-1β. Strikingly, IL-1β released from platelets mediates platelet activation cascade and promotes arterial thrombosis by acting on integrin αIIbβ3 outside to inside signal transduction (Qiao et al., 2018). We therefore set out to investigate whether IL-1β participates in the regulation of integrin αIIbβ3 via PD-L1. Our data showed that the addition of IL-1β or treatment with MDK83190 in PD-L1 KO platelets recovered its decreased platelet aggregation (Figure 4E). And the addition of IL-1β in PD-L1 KO platelets restored the speed of clot retraction and increased the clot size, while the addition of anti-IL-1β antibody in WT platelet slowed down the speed of clot retraction and decreased clot size (Figure 4F). Our results proved that PD-L1 knockout could reduce IL-1β release through the Caspase-3/GSMDE pathway to inhibit integrin αIIbβ3 activation.

### Inhibition of PD-L1 Reduces FeCl_3_-Induced Occlusion of the Carotid Artery *in vivo*

**FIGURE 5:**
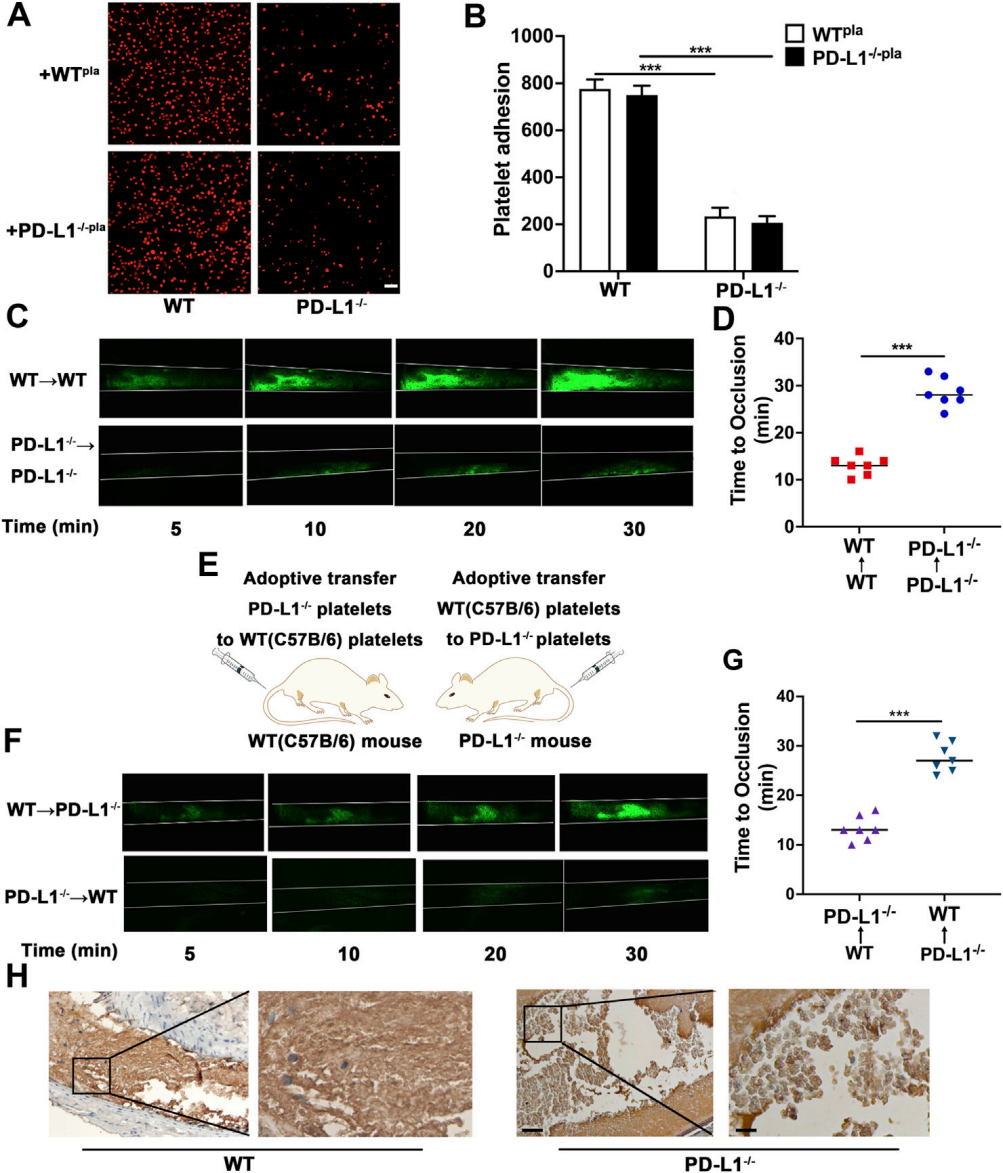
PD-L1 knockout leads to a significant decrease in platelet adhesion and inhibites carotid artery thrombosis. **(A,B)** Representative images of surface coverage by PKH26-stained platelet on a fibrinogen surface. Platelet isolated from WT and PD-L1^−/−^ mice were reconstituted with isolated poor platelet plasma from the WT or PD-L1^−/−^ mice. Place the platelet on a fibrin-coated glass slide (100 ug/ml fibrinogen, overnight at 4 °C) at 37 °C for 90 min. Washed with PBS and observed the slides with a confocal microscope. *n* = 5. Scale bar = 10 μm. **(C)** Representative fluorescence microscopy images at 5, 10, 20 and 30 min after FeCl_3_ application. Mice were subjected to FeCl_3_-induced thrombosis of carotid arteries (5% FeCl_3_, 5 min). Thrombus formation was monitored by analyzing exogenous carboxyfluorescein succinimidyl ester (CFSE)-labeled platelet accumulation by intravital microscopy and recording videos (fluorescence) of microscopic images every 2 min; *n* = 6. **(D)** Quantitative graphs of artery occlusion time. **(E)** R300 was used to eliminate platelets in PD-L1^−/−^ mice and WT mice. Adoptive transfer of PD-L1 KO platelets and WT platelets to WT and PD-L1^−/−^ mice after 12 h of R300 treatment; *n* = 5. **(F)** Mice were subjected to FeCl_3_-induced thrombosis of carotid arteries (5% FeCl_3_, 5 min); *n* = 6. Thrombus formation was monitored by analyzing exogenous CFSE-labeled platelet accumulation by intravital microscopy and recording videos (fluorescence) of microscopic images every 2 min. Representative fluorescence microscopy images at 5, 10, 20 and 30 min after FeCl_3_ application. **(G)** Quantitative graphs of artery occlusion time. **(H)** Representative immunohistological images of CD42b in carotid artery thrombus. Platelets were marked brown by CD42b antibody; *n* = 6. Bar represents = 50 μm (left); Bar represents = 10 μm (right). Data are mean ± SD. ***p < 0.001.

It is well established that platelet adhesion correlating with the platelet activation status is the necessary step of thrombosis; we reasoned that PD-L1 knockout inhibited platelet adhesion. We observed that PD-L1 KO platelets had a lower degree of adhesion on immobilized fibrinogen compared with WT mice platelets. Next, we expanded our work towards the effect of a stimulatory factor in plasma. When washed PD-L1 KO platelets were reconstituted with heterologous WT plasma and washed WT platelets were reconstituted with heterologous PD-L1 KO plasma, the adhesive response of PD-L1 KO platelet was recapitulated. This observation indicated that the reduced phenotype of PD-L1 KO platelets was intrinsic rather than secondary to activating stimuli in plasma (Figures 5A,B).

To gain deeper insights into the effect of platelet PD-L1 on thrombosis, we next assessed the time to occlusion of carotid arteries due to thrombus formation after ferric chloride (FeCl_3_)-induced injury in mice. Arterial thrombotic occlusion was significantly delayed and decreased in PD-L1^−/−^ mice after FeCl_3_-triggered vascular injury of a carotid artery (Figures 5C,D). Of note, we took advantage of adoptive transfer experiments, to confirm the necessity of platelet PD-L1 in thrombosis. Specifically, we used anti-CD42b antibodies (R300) to deplete platelets. After that, fluorescently carboxyfluorescein succinimidyl ester (CFSE) -labeled WT or PD-L1 KO platelets were infused into the thrombocytopenic mice, followed by intravital microscopy (Figure 5E). Notably, compared with PD-L1^−/−^ mice transfused with WT platelets, WT mice transfused with PD-L1 KO platelets displayed a significant reduction in the thrombus size at the site of vascular injury and manifested by a longer vessel occlusion time (Figures 5F,G). Our data indicated that inhibition of platelet PD-L1 prevents arterial thrombotic occlusion or thromboembolism. To gain deeper insights into the internal structure of the thrombus, we took advantage of immunohistochemical experiments, where platelets were labeled by Anti-CD42b. immunohistochemical figures revealed that the thrombus was mainly composed of loosely arranged RBCs and few loose platelets in PD-L1^−/−^ mice, while massively dense platelets accumulated in the entire thrombus in WT mice (Figure 5H). Our results confirmed that inhibition of platelet PD-L1 could effectively improve carotid artery thrombosis.

### The Absence of PD-L1 ameliorates Infarct Volume and Reduces Neurological Deficits

**FIGURE 6:**
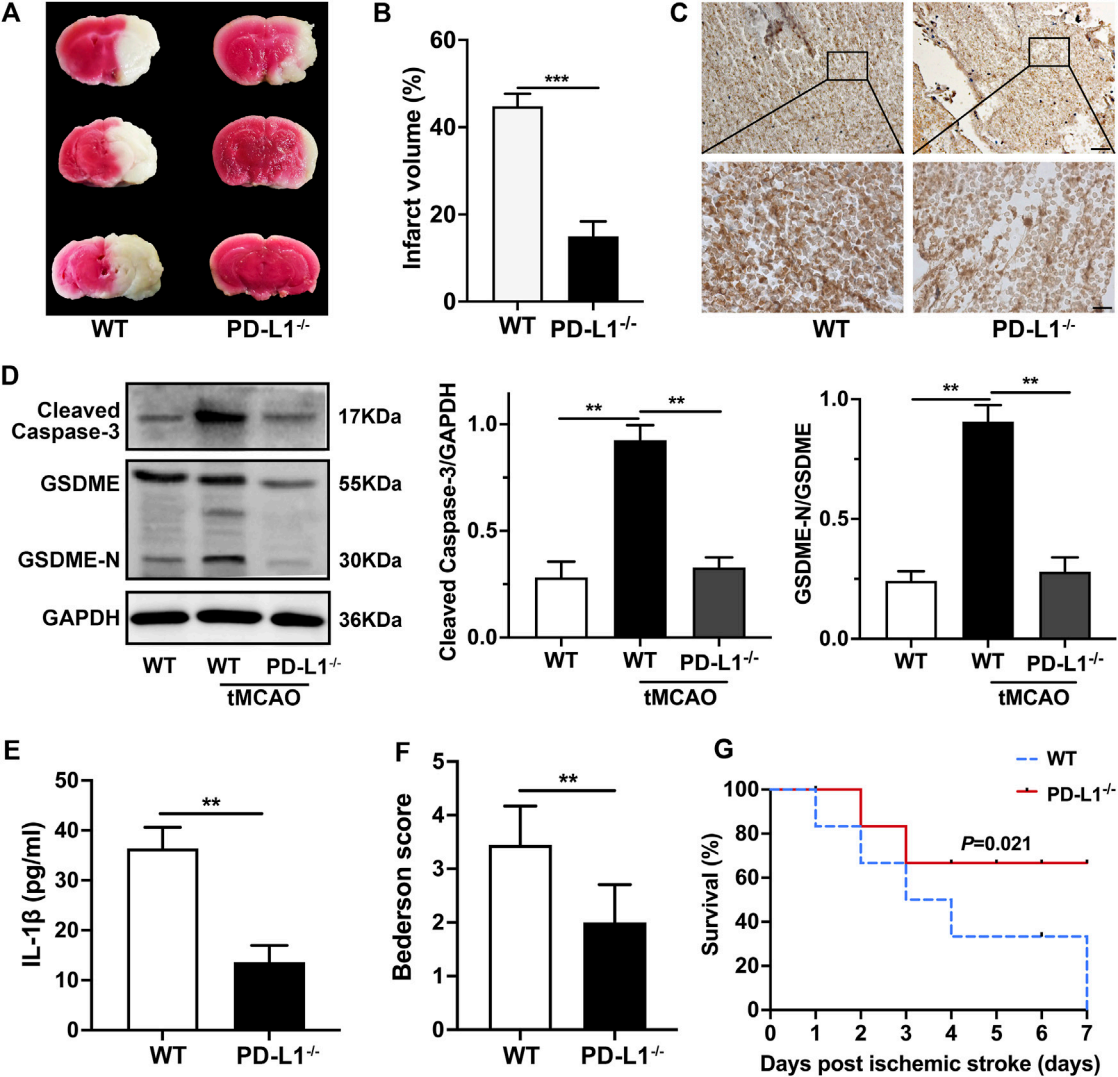
Knockout PD-L1 ameliorates ischemic stroke and improves neurological function through Caspas-3/GSDME signaling. **(A)** Representative images of three corresponding coronal sections of 2,3,5-triphenyl tetrazolium chloride–stained brains from PD-L1^−/−^ mice and WT mice 24 h after tMCAO; *n* = 7. **(B)** Brain infarct volumes of WT mice and PD-L1^−/−^ mice 24 h after tMCAO; *n* = 7. **(C)** Extract cerebral thrombus from WT and PD-L1^−/−^ mice 24 h after tMCAO. Platelets were stained with CD42b (brown). The images show a representative immunohistochemical stained section of mouse cerebral thrombosis. Bar represents = 50 μm (up). Bar represents = 10 μm (down). *n* = 6. **(D)** Representatives immunoblot of the GSDME in platelets of WT mice and PD-L1^−/−^ mice 24 h after tMCAO. GAPDH was used as loading controls. *n* = 6. **(E)** The levels of platelets IL-1β in WT and PD-L1^−/−^ mice 24 h after tMCAO were detected by enzyme-linked immunosorbent assay. *n* = 6. **(F)** Bederson score reflecting global neurological defects (0 indicates best, three indicates worst) of WT mice and PD-L1^−/−^ mice 24 h after tMCAO; *n* = 7. **(G)** Analysis of survival of PD-L1^−/−^ mice (red line) and WT mice (blue line) 7 days after ischemic stroke; *n* = 7–9. Data are mean ± SD. *p < 0.05, **p < 0.01, ***p < 0.001.

To further evaluate the impact of platelet PD-L1 on responses and consequences after ischemic stroke, we conducted the tMCAO model. And we analyzed the 2,3,5-triphenyl tetrazolium chloride staining of brain sections. Our data showed brain infarct volumes were significantly decreased in PD-L1^−/−^ mice 24 h after tMCAO (Figures 6A,B). In line with the above data, immunohistochemical analysis showed less platelet coverage in PD-L1^−/−^ mice cerebral thrombi, while in cerebral thrombosis of WT mice closely arranged more platelets (Figure 6C). Our results revealed cerebral thrombosis’s internal organization and structural features and defined the importance of platelet PD-L1 in cerebral thrombosis. Then we verified the regulation of PD-L1 on Caspase3/GSDME signaling in tMCAO mice platelet. As expected, the expression of Caspase-3/GSDME in PD-L1^−/−^ mice platelets after tMCAO was significantly lower than that in WT mice (Figure 6D). And the release of IL-1β in platelet of PD-L1^−/−^ mice after tMCAO was also markedly lower than that in WT mice (Figure 6E). PD-L1^−/−^ mice had a significantly decreased Bederson score reflecting the improvement of neurological function (Figure 6F). PD-L1^−/−^ mice further displayed significantly improved outcomes because PD-L1 knockout resulted in increased survival 7 days after tMCAO (Figure 6G).

In sum, our results showed that PD-L1 knockout in platelet reduced the release of IL-1β through regulating Caspas-3/GSDME alleviated ischemic stroke and thrombotic cerebral vascular occlusions with significantly better neurological effects and survival rates after tMCAO.

## Discussion

In recent years platelets have gained attention as essential players in cancer growth and metastasis; it has been gradually known that they also function as immune cells. Immune checkpoint therapies that target the programmed death-1 receptor ligand (PD-L1) have shown unprecedented rates of durable clinical responses in patients with various cancer types. Therefore, the growing insights into the complex interactions between cancer cells and platelets as well as cancer-cells and PD-L1 have reminded researchers to pay attention to the correlation between the three. Clemens H’s study found that platelets from non-small-cell lung cancer (NSCLC) patients expressed PD-L1, and PD-L1 could transfer from tumor cells to platelets in an integrin- and GPIbα-dependent manner in tumor immune evasion (Hinterleitner et al., 2021). Alexander B’s work showed that platelet-derived PD-L1 regulated the growth of PD-L1-negative tumors and interfered with platelet binding to PD-L1-negative cancer cells to promote T cell-induced cancer cytotoxicity (Zaslavsky et al., 2020). Sheetal B and colleagues’ study found that PD-L1 might be associated with ischemic stroke in mice. All these findings strongly suggest the correlation between PD-L1 and platelets, but the specific function of PD-L1 on platelet is unknown.

This study first evaluated that PD-L1 was a regulator of platelet-associated thrombosis. Here we presented several new vital findings that might have clinical implications. First, the absence of PD-L1 inhibited platelet activation and thrombosis. Second, PD-L1 regulated platelet integrin αIIbβ3 activation through the Caspase3/GSDME signaling. Furthermore, PD-L1 knockout improved stroke effects and ameliorated post-stroke nerve injury in mice by inhibiting platelet activation. Our study sheds light on the biological role of platelet PD-L1. PD-L1 might be a novel platelet regulator, and anti-PD-L1 might serve as a potential treatment for ischemic stroke and thrombosis prevention.

PD-L1 expression in human platelets was first reported in 2018 and is mainly located on the human platelet membrane (Rolfes et al., 2018); afterward, several studies have reported that human platelets express PD-L1 (Zaslavsky et al., 2020; Darga et al., 2021; Hinterleitner et al., 2021). A recent study reported that PD-L1 was also found in the platelet α-granules (Hinterleitner et al., 2021), which might suggest that PD-L1 also be a platelet releasates. As one of the platelet contents, α-granules could be released from activated platelets, PD-L1 located on the α-granules would be released together. Can the platelet PD-L1 could be a ligand? Previous research showed that infusion of PD-L1-overexpressing platelets could inhibit the progress and reverse the new-onset type 1 diabetes in NOD mice. PD-L1-overexpressing platelets and their released PMPs could accumulate in the inflamed pancreas and execute the immunosuppressive function (Zhang et al., 2020). What’s more, Kanikarla-Marie P et al. believed that there could be some connection between unresponsive circulating tumor cells (CTCs) and platelet counts in circulation as escape mechanisms for CTCs from immune surveillance could be achieved by being entrapped within platelet aggregates, or by expressing platelet proteins on their surface and masking themselves (Kanikarla-Marie et al., 2018), and Diem S, et al., showed that patients who had higher platelet counts showed poorer response to PD-L1 therapy (Diem et al., 2017). Functionally, PD-L1 might be a ligand. In our future studies we will further focus on the function of PD-L1 as a ligand or platelet releaser.

Integrin αIIbβ3 (GPIIbIIIa) is the most abundant integrin receptor on the platelet surface, which is transported to the platelet surface after platelet activation to amplify further platelet activation and aggregation (Calaminus et al., 2007; Blair and Flaumenhaft, 2009; Li et al., 2010). Platelets are reactivated by the direct interaction of αIIbβ3 with fibrinogen and other matrix proteins (Senis et al., 2014; Manne et al., 2015; Makhoul et al., 2020). The outside-in signaling of platelet integrin αIIbβ3 receptors is thought to be the leading cause of platelet activation and induction of thrombosis regulated by the protein tyrosine kinase (PTK) family of downstream Src family kinases (SFKs) (Hynes, 1992; Tadokoro et al., 2003). Phospholipase Cγ2 (PLCγ2) activates the serine/threonine-protein kinase C (PKC) family through Src, respectively, and promotes Ca^2+^ mobilization in platelets, mediating a cascade of platelet activation signaling (Shattil and Newman, 2004). Subsequently, the cytoskeleton domain changes induce the signal transduction of integrin from the outside to the inside, mediate the firm adhesion and aggregation of platelets to the exposed extracellular matrix via Src family kinases involved, inducing irreversible platelet aggregation and clot contraction (Obergfell et al., 2002). To investigate the role of PD-L1 in αIIbβ3 outside-in signaling, we measured platelet spreading and clot retraction. We found that PD-L1 knockout significantly decreased platelet spreading on immobilized fibrinogen and impaired clot retraction. It has been reported that PD-L1 could activate Src family kinases to suppress pain in mice (Liu et al., 2020) and recruit Src family kinases to mediate its biological roles in immune cells (Hebeisen et al., 2013). Our results proved that PD-L1 knockout significantly inhibited platelet Src and PLCγ2 expression to reduce αIIbβ3 activation.

IL-1β could increase platelet adhesion to collagen and fibrinogen (Beaulieu et al., 2014), and IL-1β could participate in the regulation of αIIbβ3 outside-in signaling in platelets (Qiao et al., 2018). Bowen Z’s study identifies GSDME as a conduit for IL-1β release independent of its ability to cause cell death (Zhou and Abbott, 2021). We found that PD-L1 KO platelets significantly reduced the release of IL-1β. GSDME, as a conduit for IL-1β release (Zhou and Abbott, 2021) mediates IL-1β release. We found that the expression of GSDME-N was significantly reduced in the PD-L1 KO platelet, suggesting the membrane-punching capacity mediated by GSDME was inhibited, resulting in reduced IL-1β release. GSDME could only be cleaved by Caspase-3, but not by Caspase 1, 4, 6, 7, 8, and 9 (Wang Y. et al., 2017). We found that the expression of Cleaved-Caspase-3 was decreased in the PD-L1 KO platelet. Activating Caspase-3 by MDK83190 could restore the activation of integrin and platelet aggregation in PD-L1^−/−^ mice. Addition of IL-1β in PD-L1^−/−^ mice platelet rescued platelet spreading and clot retraction to the level of WT platelet. Our results supported the regulator role of PD-L1/Caspase-3/GSDME in platelet integrin αIIbβ3 signaling.

As a consequence of vessel wall injury, the subendothelial matrix and collagen fibers are exposed to the flowing blood. Circulating platelets would be rapidly activated, adhere to these structures, and initiate the arrest of blood flow (Yu et al., 2019; Zhang et al., 2020; Li et al., 2021). Platelet adhesion is the first step in inducing occlusive thrombus formation in blood vessels, and platelet adhesion plays an essential role in platelet-promoting thrombosis. Therefore, through plasma washing experiments, we verified that the reduced adhesive phenotype of the PD-L1 KO platelet was intrinsic to the platelet rather than secondary to activating stimuli in plasma. FeCl_3_ to induce carotid artery thrombosis showed that carotid artery thrombosis in PD-L1^−/−^ mice was significantly attenuated. Adoptive transfer experiments were performed to confirm the findings. Infusion of WT or PD-L1 KO CFSE-labeled platelets into PD-L1^−/−^ mice or WT mice depleted platelets with R300. Infusion of WT platelets into PD-L1^−/−^ mice restored carotid artery thrombosis in PD-L1^−/−^ recipient mice to the state of WT mice. In contrast, transfusion of PD-L1 KO platelet into WT recipient mice significantly inhibited carotid artery thrombosis. Tail bleeding time in mice was detected and showed that PD-L1 knockout did not increase the tail bleeding time in mice. Our study revealed that PD-L1 might be a good target for antiplatelet thrombosis without bleeding risk.

Arterial thrombosis causes high morbidity and mortality worldwide. The most common form of arterial thrombosis is ischemic stroke, and the focus of treatment is to rapidly and effectively remove the occluded thrombus by intravenous thrombolysis or endovascular thrombectomy. However, thrombus recanalization is not always effective, which leads to failure is a lack of understanding. One possible reason could be the difference in thrombus composition, which underscores the importance of understanding the composition and structure of the tissue inside the thrombus. Several previous studies have shown that platelet-rich arterial clots are more resistant to thrombolysis by t-PA, while RBC-rich thrombi are more lysed (Tomkins et al., 2015). Therefore, thrombus components could help us better understand the pathological structure of refractory thrombus. In this study, we visualized the internal structure of the thrombus by histochemical staining. The arterial thrombus was mainly composed of RBCs in the central and platelets in peripheral regions, and RBCs-platelets in the junctional regions. The carotid artery thrombus in PD-L1^−/−^ mice showed many loosely arranged voids inside the thrombus. The platelet aggregation was reduced and replaced by loosely organized RBCs and holes. Cerebral thrombus in PD-L1^−/−^ mice also showed less platelet coverage and platelet-RBCs interactions. These results suggested that it was indeed inhibited platelet activation in PD-L1^−/−^ mice that improved the severity of thrombosis and stroke in PD-L1^−/−^ mice. Compared with WT mice, GSDME significantly decreased the release of IL-1β in the PD-L1 KO platelet 24 h after tMCAO. Our results suggested that PD-L1^−/−^ mice could inhibit integrin activation by inhibiting platelet Caspase-3/GSDME to improve the severity of the stroke.

Here, our work proved that PD-L1 could regulate platelet function and thrombosis. These findings might have positive implications for developing antiplatelet therapy in thrombosis. Treatments targeting PD-L1 showed protection against occlusive arterial thrombosis and ischemic stroke, and significantly improve outcomes. These results could have practical implications, as we provided a promising strategy for better treatment of thrombosis in the future.

## Data Availability

The raw data supporting the conclusion of this article will be made available by the authors, without undue reservation.
